# Role of the virulence plasmid in acid resistance of *Shigella flexneri*

**DOI:** 10.1038/srep46465

**Published:** 2017-04-25

**Authors:** Chang Niu, Jing Yang, Hongsheng Liu, Yong Cui, Huijie Xu, Ruifeng Wang, Xiankai Liu, Erling Feng, Dongshu Wang, Chao Pan, Wei Xiao, Xiaoqing Liu, Li Zhu, Hengliang Wang

**Affiliations:** 1 College of Life Sciences, Capital Normal University, Beijing 100048, China; 2 Beijing Institute of Biotechnology, State Key Laboratory of Pathogen and Biosecurity, Beijing 100071, China; 3 Department of Gastroenterology, Chinese PLA General Hospital, Beijing 100853, People’s Republic of China

## Abstract

Virulence plasmid (VP) acquisition was a key step in the evolution of *Shigella* from a non-pathogenic *Escherichia coli* ancestor to a pathogenic genus. In addition, the co-evolution and co-ordination of chromosomes and VPs was also a very important step in the evolutionary process. To investigate the cross-talk between VPs and bacterial chromosomes, we analyzed the expression profiles of protein complexes and protein monomers in three wild-type *Shigella flexneri* strains and their corresponding VP deletion mutants. A non-pathogenic wild-type *E. coli* strain and mutant *E. coli* strains harboring three *Shigella* VPs were also analyzed. Comparisons showed that the expression of chromosome-encoded proteins GadA/B and AtpA/D, which are associated with intracellular proton flow and pH tuning of bacterial cells, was significantly altered following acquisition or deletion of the VP. The acid tolerance of the above strains was also compared, and the results confirmed that the presence of the VP reduced the bacterial survival rate in extremely acidic environments, such as that in the host stomach. These results further our understanding of the evolution from non-pathogenic *E. coli* to *Shigella*, and highlight the importance of co-ordination between heterologous genes and the host chromosome in the evolution of bacterial species.

Bacillary dysentery, or shigellosis, is caused by bacteria belonging to the genus *Shigella*, and is the most common cause of bacterial diarrhea. *Shigella* species and *Escherichia coli* have a high degree of homology at the genomic level, with the main difference lying in the presence (or absence) of a 230-kb virulence plasmid (VP). It is generally believed that *Shigella* evolved from *E. coli* after acquiring the VP. Bacterial evolution is a dynamic process, which can be advanced by gene loss and horizontal gene transfer among distant bacterial strains. To date, whole-genome sequencing, including both VPs and chromosomes, has been completed for many strains of all four *Shigella* species^1^,^2^. This large amount of genetic information allows us to identify the continuous genetic events that led to the evolution of pathogenic *Shigella* from non-pathogenic *E. coli*. In addition, this information provides insight into changes in virulence traits among different *Shigella* strains.

Acquisition of the VP is a key step in the evolution of *Shigella* from a non-pathogenic ancestor to a pathogenic genus^3^. Studies have shown that the VPs carried by *Shigella* species are closely related to their pathogenicity. Deletion of the VP results in loss of invasion capability of the pathogen. However, *E. coli* K-12 transformed with the VP does not have the same pathogenicity profile as *Shigella* species. In addition, transformation of the *E. coli* K-12 chromosome into *S. flexneri* can lower the virulence of the pathogen^4^,^5^. As well as acquisition of the VP, the evolution of non-pathogenic *E. coli* into pathogenic *Shigella* also involved the acquisition of chromosomal virulence genes and the loss of anti-virulence genes (“black hole” genes). There may also be cross-talk between the VP and the chromosome of *S. flexneri*. For example, some functional proteins encoded by the host cell chromosome might be regulated by the VP. However, such regulatory hierarchy is relatively unstudied, with only one previous report in which a single VP-cured *S. flexneri* strain was analyzed^6^. Regardless, this initial information suggests that the co-evolution and co-ordination of chromosomes and VPs are very important steps in the evolutionary process^7^,^8^, and should be thoroughly analyzed for a more reliable and extensive understanding of the evolution of this pathogen.

Therefore, to study the influence of VPs on bacterial chromosomes at a proteomic level, we constructed VP-cured *Shigella* strains and VP-imported *E. coli* strains. The expression profiles of protein complexes in the wild-type, deletion mutant, and transformed mutant strains were determined by blue native/sodium dodecyl sulfate-polyacrylamide gel electrophoresis (BN/SDS-PAGE), and the protein monomers were analyzed by isoelectric focusing (IEF)/SDS-PAGE two-dimensional (2-D) electrophoresis. Importantly, three different *S. flexneri* strains were included in this study to gain a broader and more reliable understanding of the interaction between the VPs and the bacterial chromosomes.

## Results

### The VP regulates the expression of GadA/B

**Figure 1:**
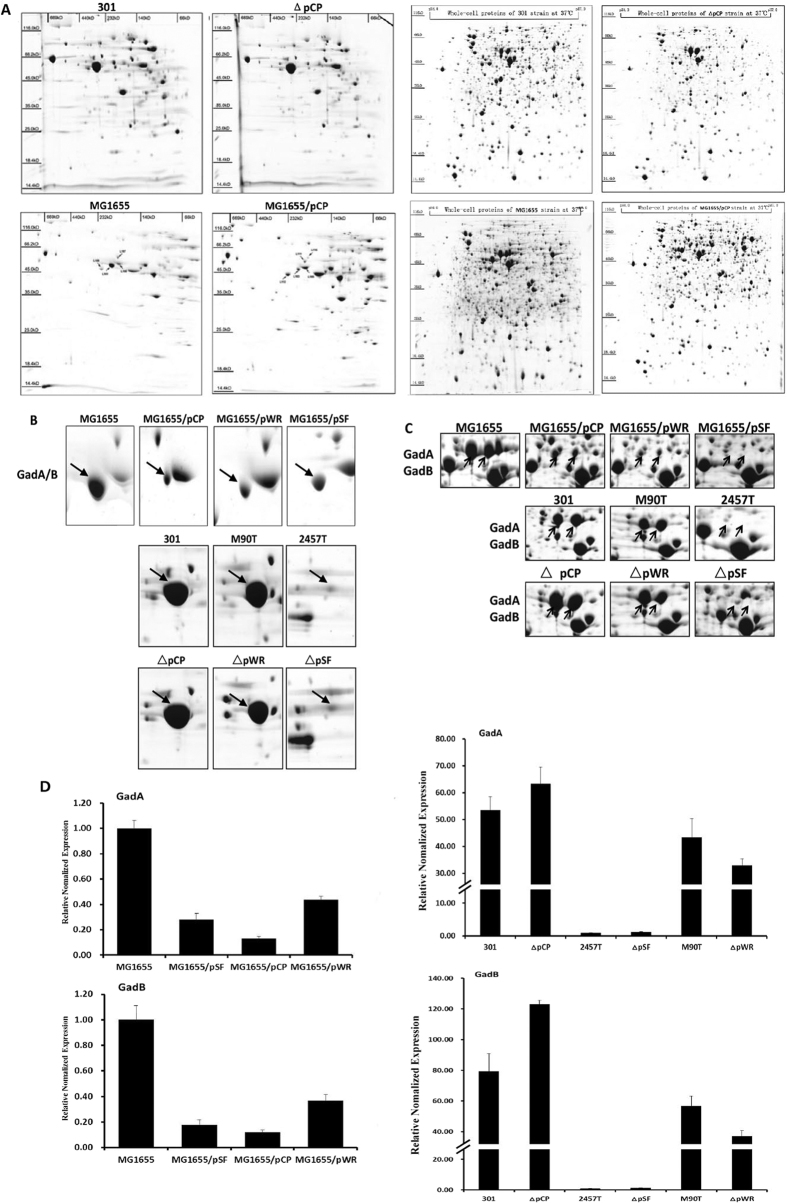
Analysis of the protein profiles of wild-type, deletion mutant, and transformed mutant strains using blue native-polyacrylamide gel electrophoresis or isoelectric focusing/sodium dodecyl sulfate-polyacrylamide gel electrophoresis. (**A**) Representative two-dimensional electrophoresis map. (**B**) Enlarged images of GadA/B protein complex. (**C**) Enlarged images of the GadA/GadB protein subunits. (**D**) Transcript levels of *gadA/B* were determined by quantitative reverse-transcriptase polymerase gel electrophoresis analysis normalized to the levels of the 16S rRNA gene in each sample.

To examine the interactions between the VP and chromosomal genes, we deleted the VPs from several *S. flexneri* strains and introduced them into *E. coli*. The interaction between the VP and the chromosome is likely to be mediated by many different proteins, which form complexes with other proteins according to their structure, dynamics, and complex physical and chemical principles to carry out their biological functions^9^,^10^. We therefore performed BN/SDS-PAGE and IEF/SDS-PAGE 2-D electrophoresis to investigate the expression profiles of protein complexes and protein monomers in the wild-type, deletion mutant, and transformed mutant strains. Figure 1A shows a representative 2-D electropherogram obtained for analysis of wild-type strain 301, its deletion mutants, and transformed mutant strains. Corresponding results for the remaining strains are shown in Supplementary Fig. S2. MALDI-TOF mass spectrometry identified the most significant differentially expressed proteins as GadA/B, which play an important role in the acid resistance of *Shigella* species. As shown in Fig. 1, the expression patterns of GadA/B protein complexes and the GadA and GadB protein monomers had similar shifting tendencies. Compared with the wild-type strain, GadA/B expression levels were significantly reduced in *E. coli* strains harboring any of the three *Shigella* VPs (pCP, pSF, and pWR). In contrast, no changes in expression were observed for the plasmid-cured *Shigella* mutants when compared with their corresponding wild-type strains (Fig. 1B,C). Interestingly, abundance of GadA/B in wild-type *S. flexneri* strain 2457 T, deletion mutant ΔpSF, and transformed mutant MG1655/pSF was extremely low (Fig. 1A,B).

To verify the proteomic data, the effects of the VP on *gadA* and *gadB* mRNA levels were quantified using quantitative real-time polymerase chain reaction (qRT-PCR) analysis. Consistent with the protein expression data, the presence of the VP was associated with reduced transcriptional levels of *gadA* and *gadB* mRNA in transformed mutant strains, but did not significantly alter transcription in VP-deficient *Shigella* strains. In addition, the expression of *gadA* and *gadB* was relatively low in 2457 T strains (Fig. 1D).

### The VP regulates acid tolerance of the host bacterium

**Figure 2:**
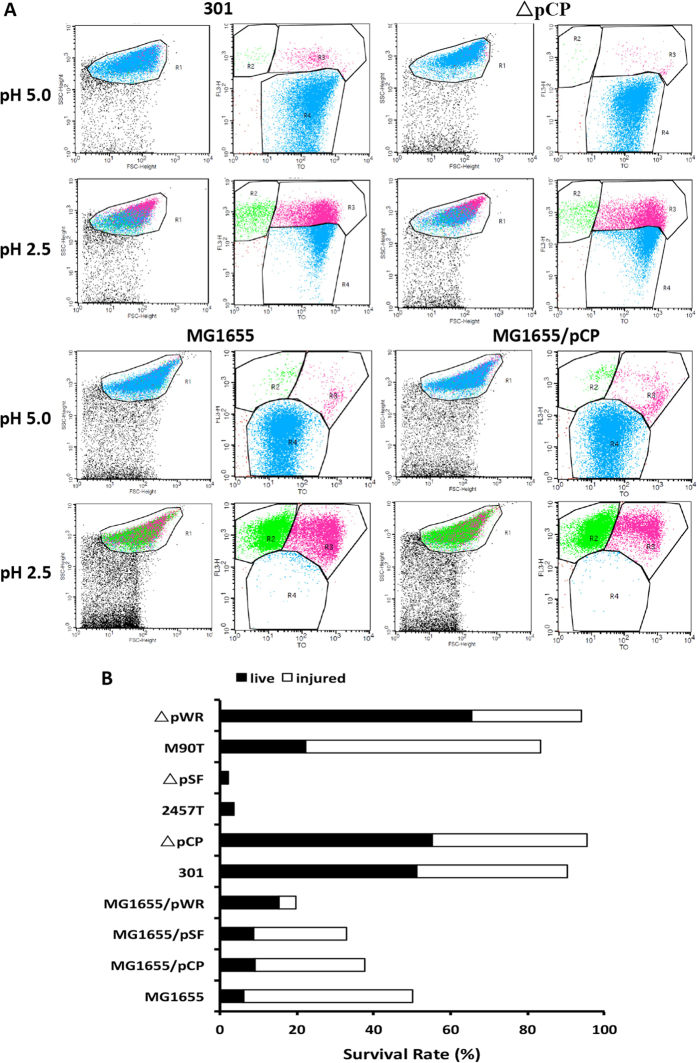
The survival rates of each strain following acid challenge analyzed by Flow cytometry. (**A**) Flow cytometry was used to provide counts of living cells before and after acid treatment, calculate the viability of bacterial cells, and then infer the strength of the acid tolerance. R1 represented the whole cells; R2–R4 were set around the dead, injured, and live bacterial populations, respectively. (**B**) Survival rates of bacterial cells were calculated following culture in acid medium. Injured cells also counted as surviving cells.

As the VP can regulate the expression of glutamic acid decarboxylase, which is the most effective acid-tolerance pathway, we examined whether the VP was associated with bacterial acid resistance. Cell survival rates of various strains were determined following growth at pH 2.5, and then compared with the survival rates at pH 5.0 using flow cytometry (injured cells were counted as surviving cells). Figure 2A is representative of results obtained for *S. flexneri* wild-type strain 301, its deletion mutants, and transformed mutant strains, while the results for the remaining strains are shown in Supplementary Fig. S3. As expected, the acid tolerance of the various strains was positively correlated with the GadA/B expression level. Strains with a higher abundance of GadA/B showed strong acid resistance, while the acid resistance of strains with lower abundance of GadA/B tended to be weaker (Fig. 2B).

### The VP regulates the expression of the AtpA/D complex but not that of protein monomers

**Figure 3:**
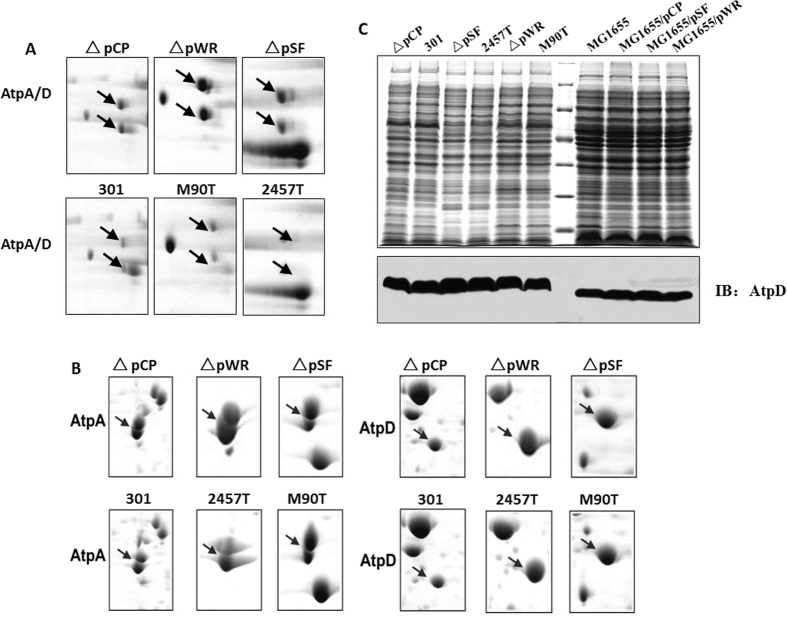
Changes of the abundance of ATP synthase subunits after the loss of VPs in Shigella strains. (**A**) and (**B**) Analysis of the AtpA/D protein complex (**A**) and AtpA (AtpD) protein subunit (**B**) from wild-type and deletion mutant *Shigella* strains using blue native-polyacrylamide gel electrophoresis or isoelectric focusing/sodium dodecyl sulfate-polyacrylamide gel electrophoresis (SDS-PAGE). (**C**) Detection of AtpD in protein samples from the wild-type and deletion mutant *Shigella* strains using western blot analysis. The samples were separated by 12.5% SDS-PAGE, and immunoblotted with anti-AtpD.

The AtpA/D protein complex is also associated with bacterial intracellular pH regulation. The γ, δ, and ε subunits of ATP synthase are difficult to detect by 2D electrophoresis as they have low molecular weights. Thus, the relative abundance of the α (AtpA) and β (AtpD) subunits is used to represent the expression of the ATP synthase complex. As shown in Fig. 3A and B, deletion of the VP greatly increased the abundance of the AtpA/D complex, while the abundance of the protein monomers remained virtually unchanged. This trend was consistent in all three of the strains analyzed. We also examined the expression of AtpD using western blot analysis, and the results mainly coincided with those generated by IEF/SDS-PAGE analysis (Fig. 3C). The expression of the AtpD protein monomer appeared to be identical in wild-type and deletion mutant strains. Therefore, the VP altered the expression of the ATP synthase complex, but had no effect on the monomers.

**Figure 4:**
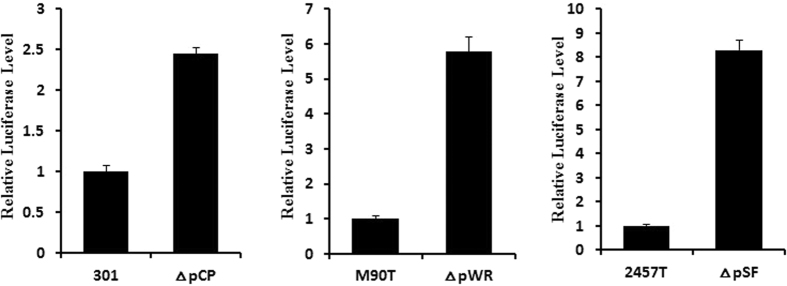
Detection of intracellular ATP levels in wild-type and deletion mutant *Shigella* strains. Bacterial samples were lysed, and the intracellular ATP level was measured in a bioluminescence assay using an ATP Assay System Bioluminescence Detection Kit.

An ATP Assay System Bioluminescence Detection Kit was then used to analyze the intracellular ATP levels of wild-type and VP-cured *Shigella* strains. According to the luminescence detection results (Fig. 4), the ATP concentrations were greatly increased in the three deletion mutant strains. Combined with the previous results of BN/SDS-PAGE 2D electrophoresis (Fig. 3A,B), we can infer that the VP reduced intracellular ATP synthesis by decreasing the expression of the ATP synthase complex.

### The VP suppressed the assembly of ATP synthase

**Figure 5:**
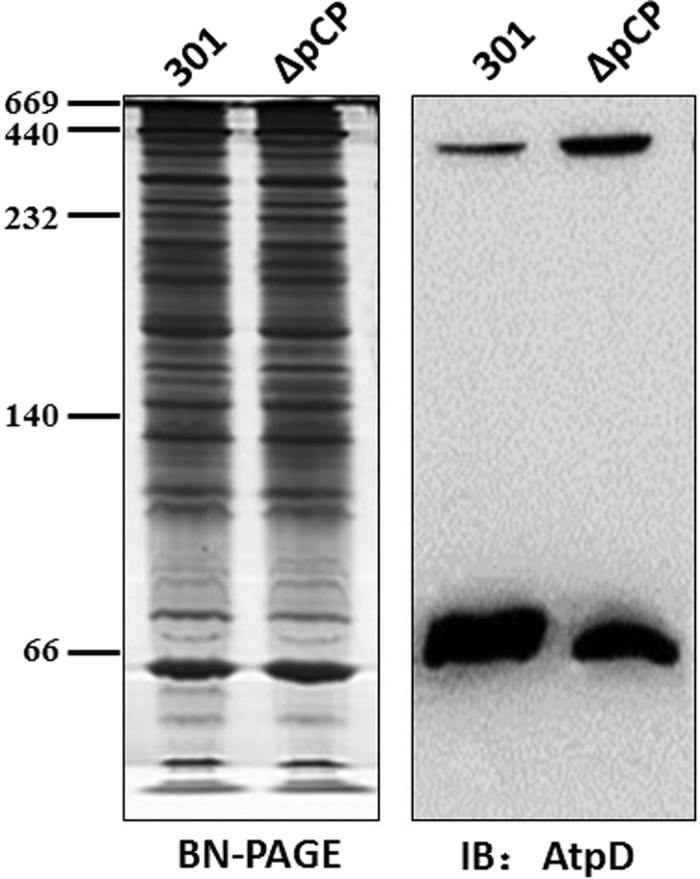
Detection of AtpD in protein complex samples using western blot. Protein complex samples were separated by 6–11% native sodium dodecyl sulfate polyacrylamide gel electrophoresis (upper panel) and then immunoblotted with anti-AtpD (lower panel).

As the levels of the ATP synthase protein complex did not correlate with the abundance of each of the protein monomers, we speculated that the VP could block the assembly of ATP synthase. To test this hypothesis, the 385-kDa ATP synthase complex and the ~50-kDa AtpD monomer were isolated simultaneously using a non-denaturing 6–11% acrylamide gradient gel. AtpD antibody analysis detected two bands (Fig. 5). The upper bands on the western blot should be protein complexes, while the lower bands may be free AtpD monomers. The abundance of the two sets of bands showed a counter-balance relationship with each other. Based on our hypothesis, removal of the VP should allow more AtpD monomers to be assembled into ATP synthase complexes because the inhibitory effect on ATP synthase assembly would be abolished. Accordingly, in deletion mutant strains, the ATP synthase complex was highly expressed, and the abundance of the AtpD monomer was lower than in wild-type strains. As a membrane protein, ATP synthase is distributed on the cellular membrane of prokaryotes^11^. Notably, the type III secretion system (T3SS), which plays a key role during the invasion of pathogens, is also located on the bacterial cellular membrane^12^. Thus, there may be spatial competition between ATP synthase and the T3SS.

Together, these findings further our understanding of the complex evolution from non-pathogenic *E. coli* to pathogenic *Shigella*, and identified a novel role for the VP in regulating acid resistance-related genes present on the chromosome. We also confirmed that glutamate decarboxylase is the primary acid-resistance mechanism in *Shigella*, and that its expression is closely correlated with the acid tolerance of these pathogens.

## Discussion

*Shigella* species are highly pathogenic, with reports that as few as 10 organisms can cause infection. With a long incubation period, the diagnosis and treatment of *Shigella* infection is even more difficult. All enteric pathogens must pass through the extremely acidic environment of the stomach before they reach the neutral environment of the intestinal lumen. *Shigella* strains are often more resistant to gastric acid than other intestinal bacteria such as *Salmonella* and *E. coli*, which may allow the survival of a small number of cells, and thus provide the opportunity for *Shigella* to invade the intestinal mucosa^13^. *E. coli* have acquired five acid resistance pathways to counteract the extreme acidity of the stomach, but there are only two main acid-tolerance pathways in *Shigella* species: the AR1 (decarboxylase independent pathway) and AR2 (glutamate decarboxylase system) pathways^14^,^15^. The glutamate decarboxylase system is particularly important for survival of extremely acidic environments^16^. Our analyses showed that GadA/B expression was significantly reduced in *E. coli* harboring the VP, but was not significantly altered in VP-cured *Shigella* strains.

The glutamate decarboxylase system encompasses three genes: *gadA, gadB*, and *gadC*. GadA and GadB have highly homologous sequences; thus, with similar structures, they can form a 330-kDa hexamer^14^. X-ray crystallography has been used to analyze the structures of GadA and GadB^17^,^18^, and each monomer comprises three domains: an N-terminal domain, a large pyridoxal-5′-phosphate-binding domain, and a C-terminal domain. Among these, the N-terminal domain is the most important as it ensures that GadA/B is bound preferentially to the intracellular membranes in a low pH environment. The pyridoxal 5′-phosphate-dependent enzyme activity converts the α-decarboxylation product of L-glutamate to γ-aminobutyric acid and carbon dioxide^19^, consuming a cytoplasmic proton in the process^20^. This pathway prevents the internal pH from decreasing to lethal levels, as protons leaking into the cell during acid stress are consumed and excreted^21^. Because GadA/B was closely related to acid resistance, we used flow cytometry to determine the survival rates of the strains under acidic stress. We confirmed that the acid tolerance of the various strains was positively correlated with GadA/B expression level.

In addition, analysis of protein expression profiles showed that deletion of the VP greatly increased levels of the AtpA/D complex, while the expression of the protein monomers remained unchanged. ATP synthesis is one of the main chemical reactions in most organisms. However, a hydrogen ion (H^+^) can be released during the synthesis of ATP from inorganic phosphate and ADP by the ATP synthase, which makes the intracellular environment even more acidic and unfavorable for bacterial survival. ATP synthase is distributed on the cell membrane in prokaryotes, and plays a key role in cellular energy exchange. ATP synthase consists of two parts. The F_1_ hydrophilic head consists of five different subunits (α, β, γ, δ, and ε), with a stoichiometry of α_3_β_3_γδε, which catalyze the synthesis of ATP from ADP. In contrast, the F_0_ hydrophobic tail consists of three subunits (a, b, and c), in a stoichiometry of ab_2_c_12_, forming an ion channel that allows the flow of protons^22^. Because ATP synthase is composed of a variety of subunits and the assembly process is relatively complex, many factors can affect its expression. BN-PAGE and western blotting analysis suggested that the VP suppressed the assembly of AtpA/D. Because the VP contains a large number of T3SS-related genes, the synthesis of these proteins and assembly of the T3SS, which is an ultra-structure across two layers of membrane, may hinder the fluidity of the bacterial outer membrane. This would be harmful for the translocation of ATP synthase subunit proteins, thereby interfering with the formation of ATP synthase complexes.

**Figure 6:**
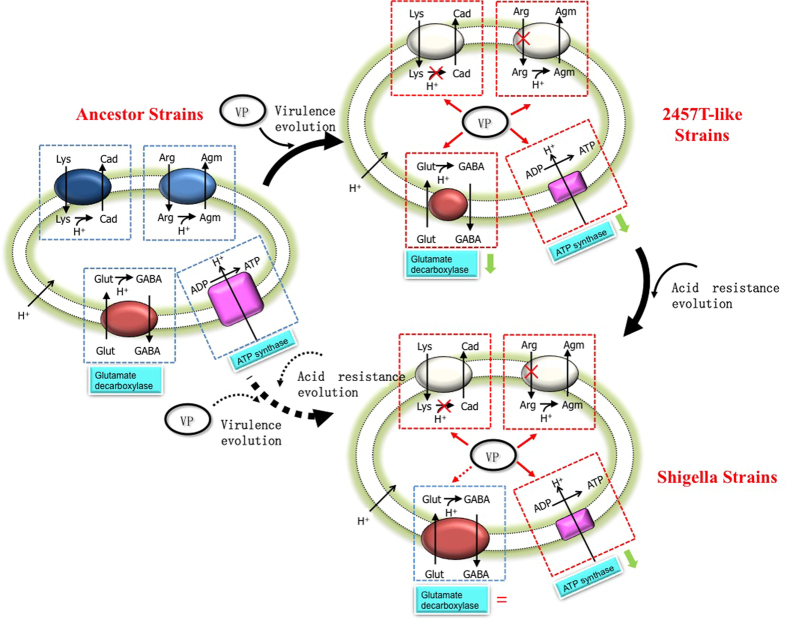
Effects of VP on acid resistance evolution from ancestor strains to *Shigella* strains. A hypothesis of the changes of glutamate decarboxylase and ATP synthase expression during the long period of co-evolution of the VP and the chromosomal genes were proposed here.

Combining all of the above results, we speculate that during the initial stages of *Shigella* evolution, the expression of glutamate decarboxylase rapidly decreased after *E. coli* acquired the VP, which decreased the acid resistance of the bacterium. However, as acid tolerance is required for intestinal bacteria to survive in the gut and colonize host cells, the decrease in acid resistance was unfavorable. In particular, it has been reported that lysine decarboxylase is absent in *Shigella*, suggesting that the glutamate decarboxylase system is the most important acid-resistance mechanism in these species^14^. Perhaps, during the long period of co-evolution of the VP and the chromosomal genes, GadA/B expression gradually increased again via some unknown mechanism, restoring acid resistance (Fig. 6). Thus, even if the VP was deleted (as in the current study), GadA/B expression levels would not be dramatically altered as a large part of this expression is no longer controlled by the VP. More simply, the acid resistance pathways of *E. coli* and *S. flexneri* developed via different evolutionary processes. In this hypothesis, *S. flexneri* strain 2457 T, which showed lower levels of GadA/B expression, may represent a kind of intermediate strain in the evolution of *Shigella*. Despite this decreased GadA/B expression, *S. flexneri* strain 2457 T is a clinical isolate that was responsible for a dysentery epidemic. The survival advantage of 2457 T might come from the R27 plasmid, which contains multiple antibiotic resistance genes that would aid in the survival of this pathogen in a clinical setting.

In the case of ATP synthase, the release of H^+^ during the synthesis of ATP from inorganic phosphate and ADP, resulting in an increase in the acidity of the intracellular environment (Fig. 6), may be a compensatory mechanism that has evolved during the long evolutionary period as an attempt to maintain the acquired virulence after the partial loss of acid tolerance^23^. Moreover, after a bacterium enters the host, its metabolism will be slowed (except for the synthesis of invasion- and virulence-related proteins) in an attempt to resist the extremely acidic environment of the stomach and evade the host immune response^6^. Its ATP requirements also decrease accordingly, meaning that high levels of ATP synthase are not needed. Taken together, these findings suggest that the VP decreases the production of ATP synthase, which could aid in the invasion of host cells by the pathogen.

## Methods

### Bacterial strains and growth conditions

*E. coli* strain DH5α was used for plasmid construction, and was maintained on Luria-Bertani (LB) agar or broth (Difco) at 37 °C. Wild-type *S. flexneri* serotype 2a strains 301 and 2457 T, and serotype 5a strain M90T, were grown on tryptic soy agar (Difco) containing 0.01% (w/v) Congo red or in LB broth at 30 °C and 37 °C. When necessary, 50 μg ml^−1^ nalidixic acid, 50 μg ml^−1^ streptomycin, or 30 μg ml^−1^ chloramphenicol were added to the growth media.

### Construction of transconjugant and mutant *S. flexneri* strains

As shown in Supplemental Fig. S1, the VP gene fragment *virG*, amplified from *S. flexneri* strain 301 using primers virGp1/virGp2 (listed in Supplemental Table S1), was ligated into chloramphenicol resistance *pir*-dependent suicide vector pXL275, generating recombinant plasmid pXL275-virG. pXL275-virG was transformed into *E. coli* S17-λ*pir*, and then conjugated into *S. flexneri* strain 301 according to the method of Klümper *et al*.^24^. Following homologous recombination into the VP at the *virG* site, the resulting VP contained the chloramphenicol resistance marker. Using helper plasmid pRK2013, the recombinant plasmid was conjugated into *E. coli* strain MG1655, as described previously^25^. Resulting transconjugants were named MG1655/pCP, and were isolated as pure cultures for use in further analyses. Two further transconjugant strains, named MG1655/pSF and MG1655/pWR and harboring the VPs from *S. flexneri* strains 2457 T and M90T, respectively, were prepared using the same method. VP-cured strains ΔpCP, ΔpSF, and ΔpWR were constructed using plasmid incompatibility methods^6^.

### Two-dimensional polyacrylamide gel electrophoresis and data analysis

Preparation of whole-cell protein extracts (complex and monomer samples) and 2-D polyacrylamide gel electrophoresis analysis (BN-PAGE and IEF/SDS-PAGE) were performed as previously described^26^.

### Protein identification by matrix-assisted laser desorption/ionization-time of flight tandem mass spectrometry (MALDI-TOF/TOF)

All of the protein spots were analyzed by MALDI-TOF/TOF mass spectrometry. The protein spots were carefully excised from the gel, destained using destaining solution (50% methyl cyanide, 25 mM acid ammonium carbonate), and then digested for 13 h with sequencing grade modified trypsin (Roche, USA). Peptides from digested proteins were used for MALDI-TOF/TOF analysis. The MALDI-TOF mass spectrometry measurement was performed on a Bruker Ultraflex^III^ MALDI-TOF-MS instrument (Bruker Daltonics, Germany), operating in reflectron mode with 20 kV accelerating voltage and 23 kV reflecting voltage. A saturated solution of α-cyano-4-hydroxycinnamic acid in 50% acetonitrile and 0.1% trifluoroacetic acid was used as the matrix. A 1-μl volume of the matrix solution and the sample solution at a ratio of 1:1 was applied to the Score384 target well. The SNAP algorithm (S/N threshold: 5; quality factor threshold: 30) in FlexAnalysis 2.4 was used to select the 150 most prominent peaks in the mass range m/z 700–4000. The subsequent tandem mass spectrometry (MS/MS) analysis was performed in a data-dependent manner, and the 10 most abundant ions were subjected to high energy collision-induced dissociation analysis. The collision energy was set to 1 keV, and nitrogen was used as the collision gas.

### Data interpretation and database searching

To deal with one peptide mass fingerprinting and multiple TOF/TOF spectra from one sample as a single combined dataset, the raw data were first merged into one Mascot generic format file using Biotools v3.0 software, and then searched using Mascot 2.1 (Matrix Science Ltd.) against the *S. flexneri* 2a 301 database. The search included all predicted open reading frames on both the chromosome (GenBank GI: 344915202) and virulence plasmid pCP (GenBank GI: 18462515) of *S. flexneri* 2a 301 to eliminate redundancy resulting from multiple members of the same protein family, and the results were checked against the NCBInr database (version 20061021, 4,072,503 sequences) to eliminate known contaminants. The search parameters were: trypsin digestion with one missed cleavage; carbamidomethyl modification of cysteine as a fixed modification and oxidation of methionine as a variable modification; peptide tolerance maximum, ±100 ppm; MS/MS tolerance maximum, ±0.6 Da; peptide charge, +1; monoisotopic mass. Scores greater than 21 were considered significant (P < 0.05) for a local MS/MS search. For unambiguous identification of proteins, more than five peptides must be matched.

### RNA extraction and qRT-PCR analysis

Total RNA extraction and qRT-PCR analysis were performed as described previously^26^. Gene-specific primers (Supplemental Table S1) were designed using Primer Premier 5.0 software (Premier Biosoft). Relative amounts of cDNA were normalized to the amounts of 16 S rRNA cDNA in each sample.

### Acid tolerance assay

Bacteria were grown to stationary phase at 37 °C in LB medium adjusted to pH 5.0. A 1-ml aliquot of culture was then centrifuged for 5 min at 2000 × *g*, and the resulting pellet resuspended in 1 ml of LB medium adjusted to either pH 2.5 or pH 5.0^27^,^28^. The suspension was then incubated for 30 min at 37 °C with shaking at 220 rpm. Bacteria were collected by centrifugation as described above, and washed three times with PBS. Cells were then stained for 15 min using a BD cell viability kit, and measured using a BD FACS flow cytometer. The data were analyzed using CellQuest software.

### Western blot analysis

The BN- and SDS-PAGE gels were transferred to polyvinylidene difluoride membranes at 15 V for 1.5 h. The membranes were blocked with 10% (w/v) skim milk powder in TBS (100 mM Tris-HCl, pH 7.5, 0.9% (w/v) NaCl) containing 0.1% (v/v) Tween 20 (TBST) for 1 h. Membranes were then incubated in anti-AtpD antibody (Abmart Corp.) diluted in TBST for 1–2 h at room temperature, or at 4 °C overnight, at the recommended concentration, followed by detection using ECL reagents (Thermo) and manual film development.

### ATP assay system

The intracellular ATP levels of the wild-type and mutant *Shigella* strains were measured using an ATP bioluminescence assay kit (Promega, USA) as per the manufacturer’s protocol. Strains were grown in LB broth to stationary phase, diluted in dilution buffer, and lysed using the provided cell lysis reagent. After addition of the luciferase reagent, the light signal was detected. All measurements were within the linear range as determined for an ATP standard curve.

## Additional Information

**How to cite this article**: Niu, C. *et al*. Role of the virulence plasmid in acid resistance of *Shigella flexneri. Sci. Rep.* **7**, 46465; doi: 10.1038/srep46465 (2017).

**Publisher's note:** Springer Nature remains neutral with regard to jurisdictional claims in published maps and institutional affiliations.

## Supplementary Material

Supplementary Data
