# Supplementary material for: Role of the virulence plasmid in acid resistance of Shigella flexneri
Source: Sci Rep. 2017 Apr 25;7:46465. doi: 10.1038/srep46465 (PMC5404508; doi:10.1038/srep46465)
Supplement: Supplementary Data [file srep46465-s1.doc]

**Role of the virulence plasmid in acid resistance of *Shigella flexneri***

Chang Niu^1, 2‡^, Jing Yang^3‡^, Hongsheng Liu^2^, Yong Cui^2^, Huijie Xu^2^, Ruifeng Wang^1^, Xiankai Liu^2^, Erling Feng^2^, Dongshu Wang^2^, Chao Pan^2^, Wei Xiao^1^, Xiaoqing Liu^1^, Li Zhu^2^*, Hengliang Wang^2^*

**Supplementary data**


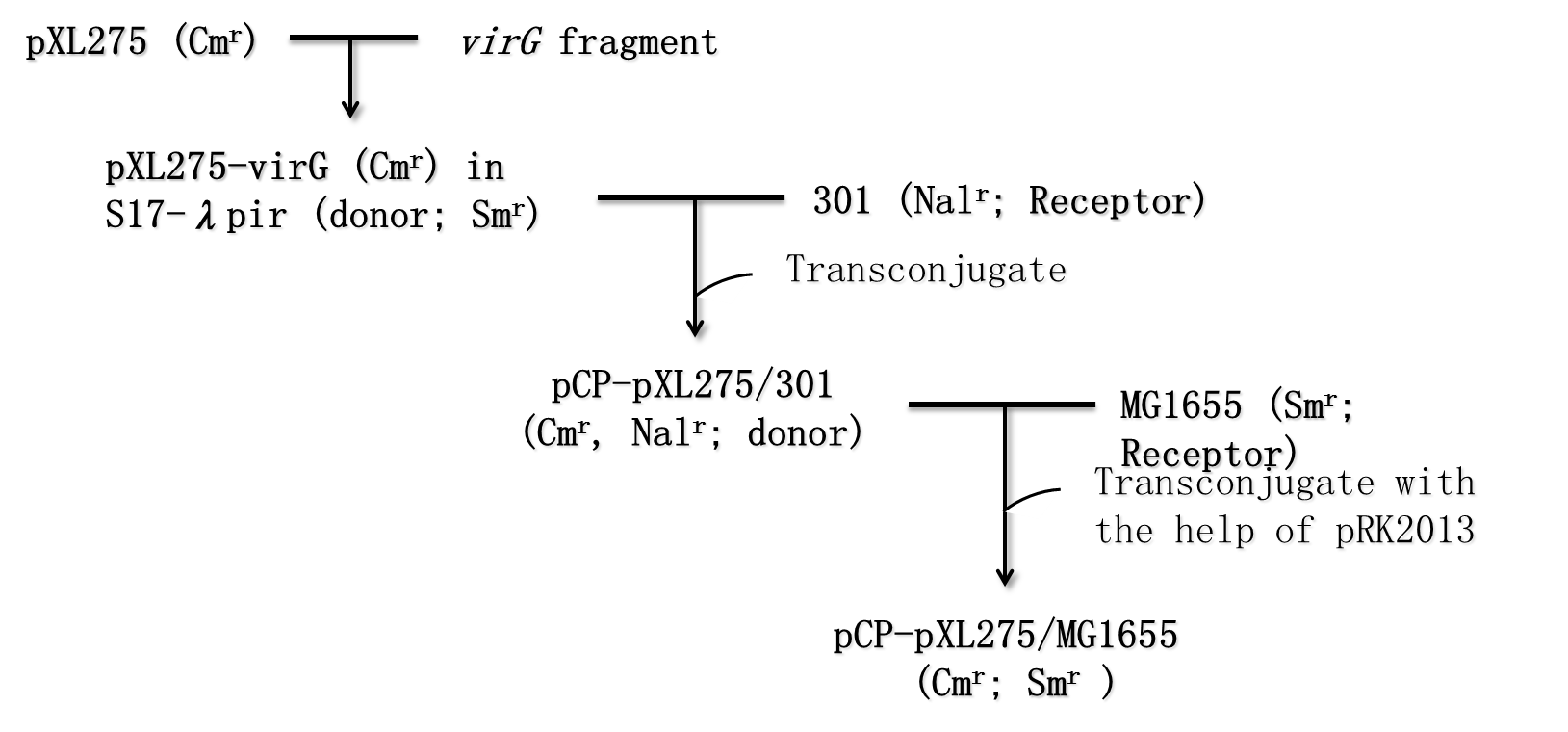


**Figure S1. Construction of the MG1655/pCP transconjugant.** Cm^r^, chloramphenicol resistance; Nal^r^, nalidixic acid resistance; Sm^r^, streptomycin resistance.


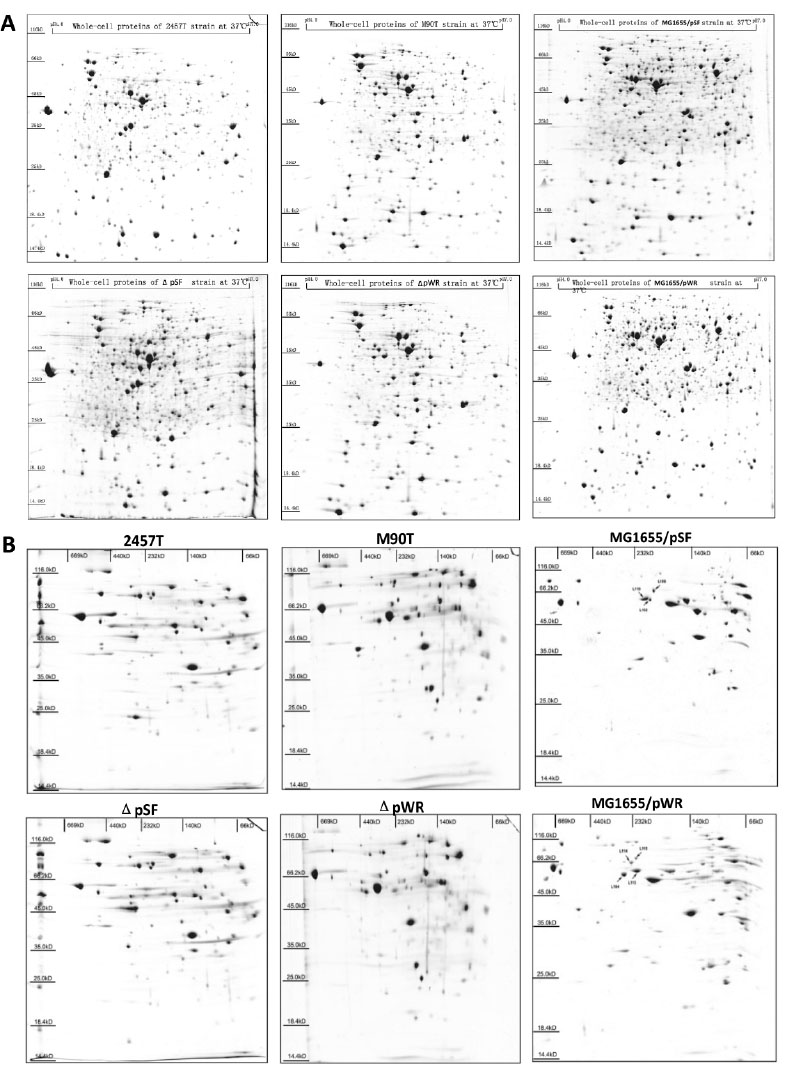


**Figure S2. Analysis of the protein profiles of wild-type, deletion mutant and transformed mutant strains using blue native-polyacrylamide gel electrophoresis or isoelectric focusing/sodium dodecyl sulfate-polyacrylamide gel electrophoresis. (a)** Electrophoresis map of protein subunits. **(b)** Electrophoresis map of soluble protein complexes.


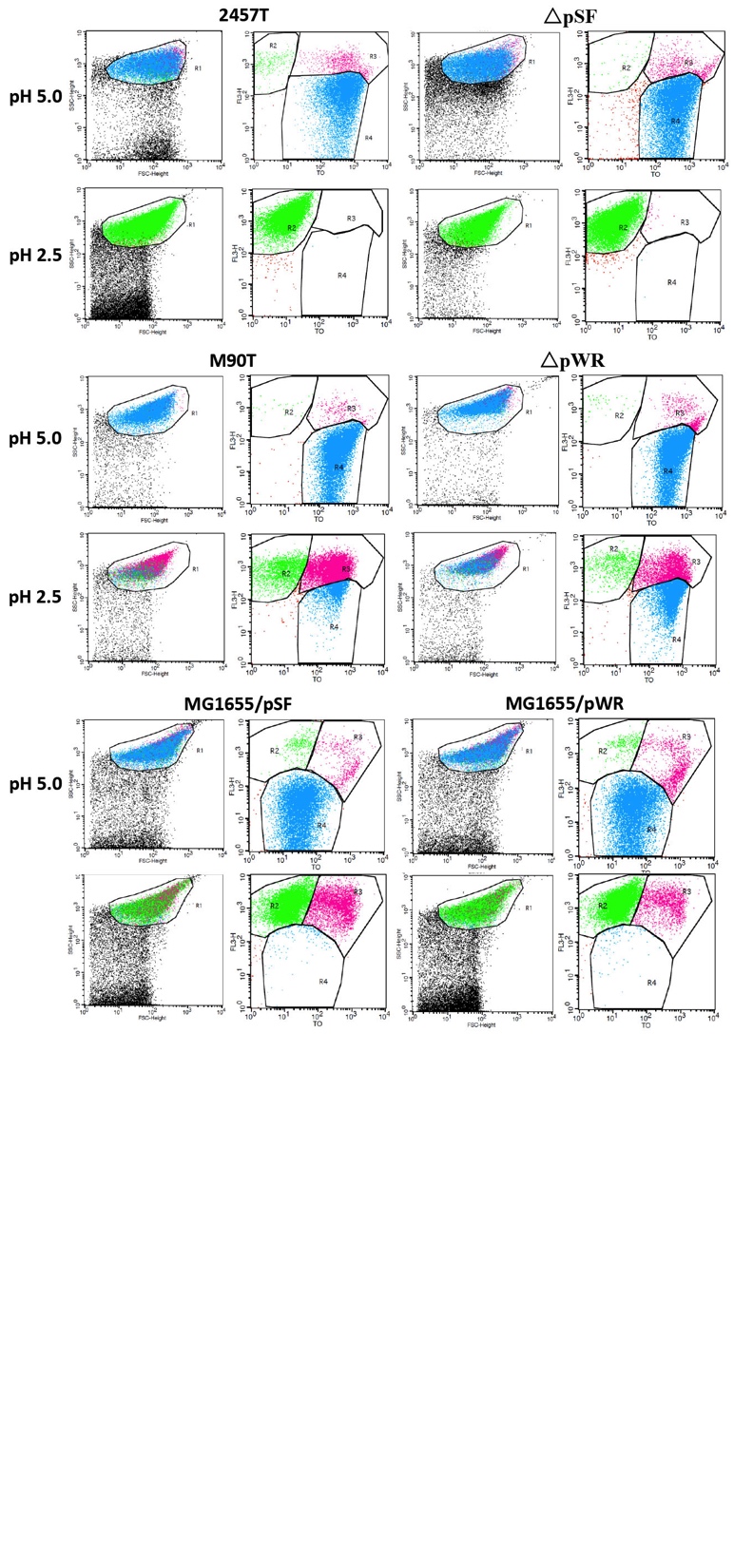


**Figure S3. The** **survival rates of each strain following acid challenge analyzed by Flow cytometry.**  Flow cytometry was used to provide counts of living cells before and after acid treatment, calculate the viability of bacterial cells, and then infer the strength of the acid tolerance. R1 represented the whole cells; R2-R4 were set around the dead, injured, and live bacterial populations, respectively.
